# Osteoid osteoma of the rib masquerading as pain due to trauma: Removed by rib resection using preoperative CT-scan guidance

**DOI:** 10.1016/j.ijscr.2023.107877

**Published:** 2023-01-09

**Authors:** Salem M. Tos, Afnan W.M. Jobran, Anas Alasafrah, Motaz Natsheh, Yousef Abu Asbeh

**Affiliations:** a Faculty of Medicine, Al Quds University, Jerusalem, Palestine; b General Surgeon, Al Ahli Hospital, Hebron, Palestine; c Pathology department, Al Ahli hospital, Hebron, Palestine; d Thoracic surgery unit, Al Ahli Hospital, Hebron, Palestine

**Keywords:** Osteoid osteoma, Trauma, Bone tumor, NSAID

## Abstract

Osteoid osteomas (OO) are benign bone tumors that are prevalent in young adults. The typical clinical picture of the disease is pain that worsens at night, which may be alleviated by Non-Steroidal Anti Inflammatory Drugs (NSAIDs). The most common imaging finding of OO is a lytic lesion, known as a nidus, with variable intralesional mineralization, accompanied by bone sclerosis, cortical thickening and surrounding bone marrow edema, as well as marked post-contrast enhancement. The most commonly affected sites are the long bones of the lower limbs, but the ribs are rarely reported sites. The present study describes a case of osteoid osteomas located in the rib which was removed by rib resection using CT-scan guidance.

## Highlights

• Osteoid osteoma (OO) is a benign bone tumor and the most commonly affected sites are the long bones of the lower limbs, but the ribs are rarely involved.
• Careful history taking, meticulous physical examination, and investigations are necessary.
• Prompt treatment should be addressed in a proper way to stabilize the patient and avoid mortality.

## 1 Introduction

Osteoid osteoma (OO) is a benign bone tumor, which was first reported by Jaffe in 1935 [1] in a series of five cases; mostly affects 5–25 years of age with male predominance [2]. OO is a common tumor, accounting for 3 % of all bone neoplasms and 10–15 % of benign lesions [2], [3], [4], [5], [6].

The typical clinical picture of the disease is pain that worsens at night, which may be alleviated by Non-Steroidal Anti Inflammatory Drugs (NSAIDs) [5], [6]. OO is highly vascularized and innervated [7], and the underlying cause of pain seems to be related to high prostaglandin levels (100–1000 × higher than normal), with prostaglandin E2 being the main subtype. In addition, these prostaglandins are responsible for vasodilatation and edema formation in the surrounding bone marrow and soft tissues [5], [6].

The most commonly affected sites are the long bones of the lower limbs, but the ribs are rarely involved. OO is diagnosed by both clinical and imaging findings. A biopsy is recommended, especially for lesions with an atypical presentation, even though it can be non-diagnostic in nearly one-third of cases [8]. OO regressed within 6–15 years, but this period can be further reduced to 2–3 years by using nonsteroidal anti-inflammatory drugs (NSAIDs) [6]. Due to the adverse effects related to the prolonged use of these medications, it is reserved for exceptional situations only, and for this reason, the more commonly used treatment option is surgical resection [9].

Herein, we report a rare case of a 13-year-old male diagnosed with osteoid osteoma (OO) of the ninth rib after presenting as continuous chest pain that is worst at night with a known history of trauma at the same site of the tumor. This work has been reported in line with the SCARE criteria, which are used by authors, journal editors, and reviewers to increase the robustness and transparency in reporting surgical cases [15].

## 2 Case presentation

This is a 13-year-old male known case of asthma, referred to the thoracic surgery department complaining of chest pain for two months duration. The pain was primarily right-sided, anterolateral aspect of the lower chest. It was continuous all over the day, and become more severe at night, measured at 07/10 according to the visual analog scale (VAS). The pain was refractory to Non-Steroidal Anti Inflammatory Drugs (NSAIDs) and acetaminophen prescribed by his family physician. There were no associated symptoms including cough, shortness of breath, or increased sputum.

**Fig. 1:**
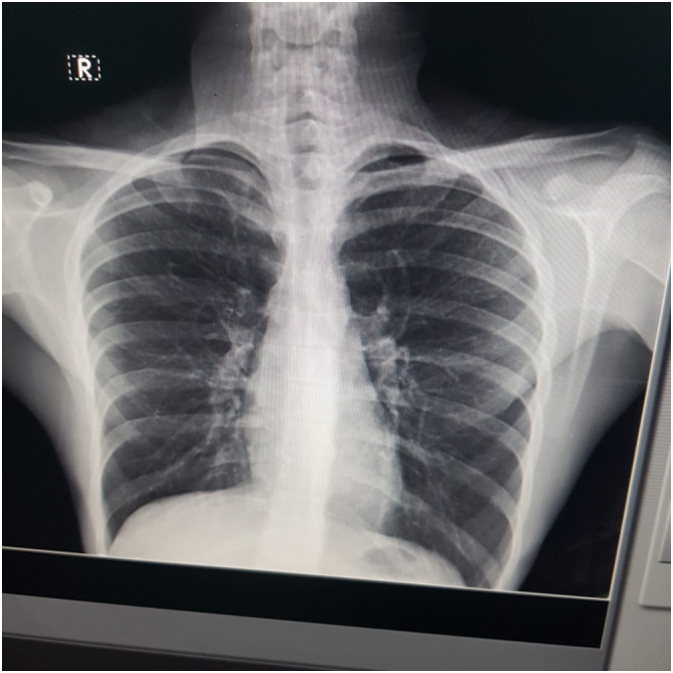
Chest X-ray performed one year ago, showed no obvious abnormalities.

One year ago, there was chest trauma over the same location. At that time, the patient was seen in the emergency department (ED) with 5/10 right-sided chest pain. After that, a Chest X-ray was performed and showed no abnormalities (Fig. 1). The patient was sent home with analgesics with the resolution of his pain in the following few weeks.

Upon examination, he was in a good general condition and normal vital signs. A fixed tenderness point was identified on the anterolateral aspect of right lower chest over the ninth rib at the level of the anterior axillary line. No associated erythema or increase in temperature. Chest bulge or deformity was also absent.

**Fig. 2:**
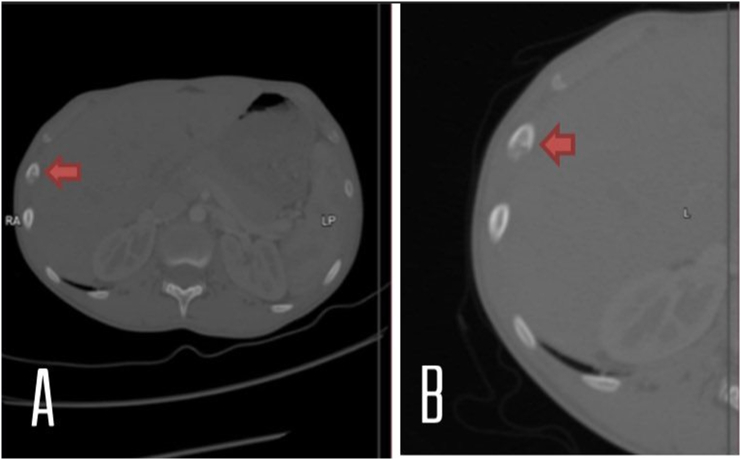
Preoperative computed tomography. Axial view (A) and zoomed in Axial view (B). A well-demarcated 5-mm osteolytic lesion with central calcification can be observed in the right 9th rib without any periosteal reaction.

The CT scan (Fig. 2) showed a well-demarcated 1-cm osteolytic lesion with central calcification in the right 9th rib. The patient's case was discussed in a multidisciplinary team meeting, rib resection was a unanimous decision. In pre-operative planning, we decided to mark the lesion with CT guidance before surgery, to be able to locate the lesion properly.

### 2.1 Intraoperative

Before surgery, the operating thoracic surgeon joined the interventional radiology team (IR) to mark the lesion, the lesion was identified and marked using a skin marker, the lesion itself was marked, and 4 cm margins were identified medially and laterally to the lesion.

Under general anesthesia, Double lumen intubation was performed with an isolate of the left lung. Arterial line and Folly's catheter were inserted, and a thoracic epidural catheter was applied for postoperative pain management. The patient was placed in the left lateral decubitus position, an anterolateral thoracotomy was performed. After identifying the nidus with a marker using CT scan guidance, a right-sided chest 10-cm incision parallel to the 9th rib was made. Complete resection of the lesion with a wide, clear safe margin was followed which includes about 4 cm medial and lateral to the lesion. In addition, the overlying serratus anterior muscle was also resected, because it was invaded by the lesion too, with attention paid to avoid an injury to the neurovascular bundle. A 20-French straight chest tube was placed and secured. The surgical incision was closed in layers. Complications were not observed during the operation. The bone specimen was sent for histological study.

### 2.2 Histopathological studies

**Fig. 3:**
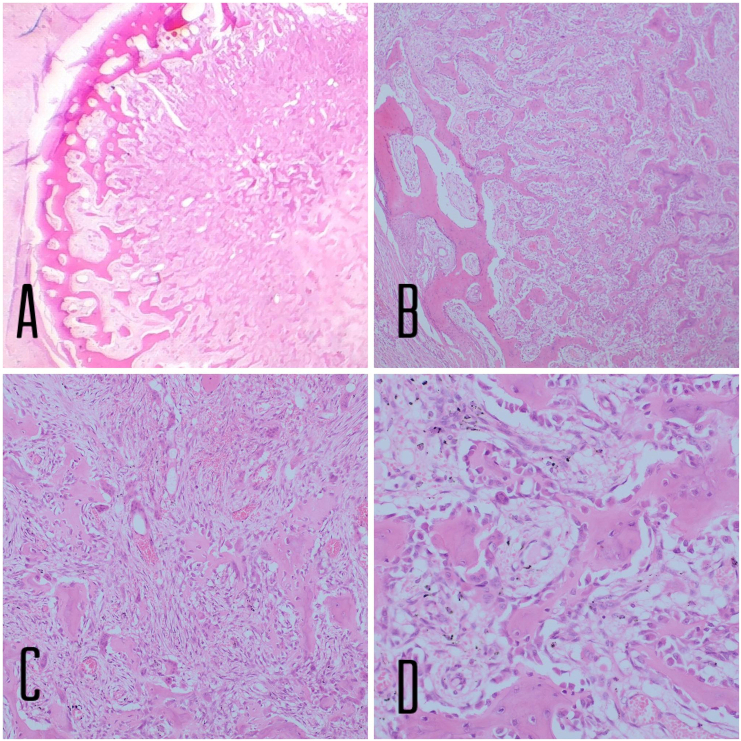
The tumor is well circumscribed and formed of haphazardly arranged trabeculae of woven bone, which rimmed by osteoblast in a loose vascularized stroma. H&E, 40×(A), 100×(B),200×(C) and 400×(D).

The tumor is well circumscribed and formed of haphazardly arranged trabeculae of woven bone, which rimmed by osteoblast in a loose vascularized stroma (Fig. 3; stain, hematoxylin and eosin), consistent with osteoid osteoma (OO).

**Fig. 4:**
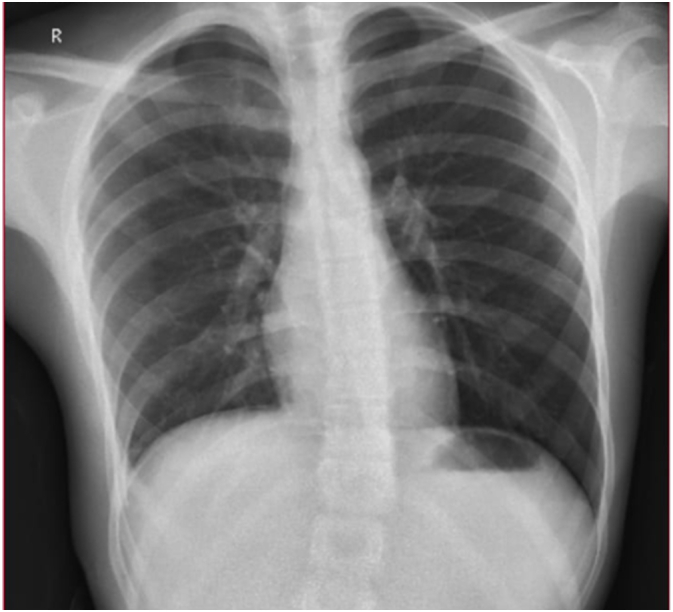
Post-operative day one, chest X-ray showed no abnormalities.

Complete pain relief associated with the nidus resection was achieved from the first postoperative day, Chest X-ray performed after operation showed no abnormalities (Fig. 4). After that, he was discharged home on postoperative day 2 after chest tube removal.

The patient is currently in good health condition with complete disappearance of chest pain after one month of the operation and does not present any complications for the long-term follow-up.

## 3 Discussion

Of all benign bone tumors, osteoid osteoma (OO) makes up about 10 % to 15 % of the total. It primarily affects young people, primarily in a male between the ages of 5 and 25 [2]. In large series, OO occurs in 6 to 9 % of people over age 40, which is not uncommon [2]. An OO is a tiny, heavily vascularized bone lesion with varying amounts of woven and osteoid bone, as well as osteoblasts that create irregular trabeculae, osteoclasts, and numerous dilated blood vessels [2]. Although the nidus tumor itself does not infiltrate nearby bone, it does cause hyperostosis and bone marrow edema [2], [3]. In some situations, specific stains inside the nidus itself can be used to show the existence of nerve fibers near the blood arteries.

Only 5–7 % of all primary bone neoplasms are rib-specific primary tumors [10]. A benign primary bone tumor with unclear pathophysiology is an osteoid osteoma (OO). With a high preference for long bones, this happens in patients throughout the first two decades of life in roughly 60 to 75 % of instances. The skull, jawbones, innominate bones, and ribs are examples of flat bones that are rarely documented [11]. Metastases and myelomas are the two tumor forms that afflict the ribs most frequently. Rib primary tumors are not prevalent. Consequently, a tumor's placement within the rib may aid in establishing a differential diagnosis. Rib sarcomas are more likely to cause pain sensations than cartilaginous tumors, which usually develop near the costochondral junction [12].

The diagnosis of OO is made using a mix of common clinical images and imaging results. The primary nocturnal symptom of this illness is pain, which can be treated with salicylates and non-steroidal anti-inflammatory medications (NSAIDs). The nidus can be hidden on radiographs; hence the CT is seen to be the best modality for OO. The central calcification is typically regular and centered, but it can also be punctate, amorphous, or ring-like. A “vascular groove” or “CT vessel” sign that corresponds to larger vessels that emerge from the periosteum to irrigate the hypervascular nidus can be seen on CT scans [13]. It is indicated by low-density grooves that enter the nidus.

For the treatment of OO, both conservative and surgical methods have been reported. A long-term NSAID regimen is used as conservative treatment, and in some circumstances, it is said to help people feel less pain. Incomplete nidus excision could result in local recurrence, according to the general principle for surgical therapy of OO. When the afflicted bone is not subcutaneous or when the visual localization of the nidus is technically challenging, a full resection of the nidus by open surgery may result in additional harm to the bone and soft tissues surrounding the lesion.

Although it is simple to access the ribs, the reported lengths of en bloc nidus resection ranged from 5 to 9.5 cm, which is much wider than the nidus's size [14].

## 4 Conclusion

This case study demonstrates that osteoid osteoma of the rib should indeed be recognized in cases of any painful rib without such a recent history of trauma and that total surgical excision, when possible, is a safe and successful treatment for this extremely uncommon bone tumor. In order to reduce the loss of respiratory function, CT-guided operations should be taken into consideration as the preferred course of treatment for OO of the rib.

## Ethical approval

Informed consent was signed from the patient for publication.

## Authors' contributions

Study concept or design: Yousef Abu Asbeh

Writing the manuscript: Salem M. Tos, Afnan W.M. Jobran, Anas Alasafrah and Motaz Natsheh

Review & editing the manuscript: Salem M. Tos, Afnan W.M. Jobran

## Content

Written informed consent was obtained from the patient and his parents for publication of this case report and accompanying images. A copy of the written consent is available for review by the Editor-in-Chief of this journal on request.

## Guarantor

Dr. Yousef Abu Asbeh.

## Registration of research studies

Not applicable.

## Declaration of competing interest

The authors declare no conflicts of interest.
